# Outcomes after Surgical Treatment of Metastatic Disease in the Adrenal Gland; Valuable for the Patient?

**DOI:** 10.3390/cancers14010156

**Published:** 2021-12-29

**Authors:** Madelon J. H. Metman, Charlotte L. Viëtor, Auke J. Seinen, Annika M. A. Berends, Patrick H. J. Hemmer, Michiel N. Kerstens, Richard A. Feelders, Gaston J. H. Franssen, Tessa M. van Ginhoven, Schelto Kruijff

**Affiliations:** 1 Department of Surgical Oncology, University of Groningen, University Medical Center Groningen, Hanzeplein 1, 9713 GZ Groningen, The Netherlands; M.j.h.metman@umcg.nl (M.J.H.M.); a.j.seinen@umcg.nl (A.J.S.); p.h.j.hemmer@umcg.nl (P.H.J.H.); 2 Department of Surgical Oncology and Gastrointestinal Surgery, Erasmus MC Cancer Institute, Doctor Molewaterplein 40, 3015 GD Rotterdam, The Netherlands; c.vietor@erasmusmc.nl (C.L.V.); g.franssen@erasmusmc.nl (G.J.H.F.); t.vanginhoven@erasmusmc.nl (T.M.v.G.); 3 Department of Endocrinology, University of Groningen, University Medical Center Groningen, Hanzeplein 1, 9713 GZ Groningen, The Netherlands; m.a.berends@umcg.nl (A.M.A.B.); m.n.kerstens@umcg.nl (M.N.K.); 4 Department of Endocrinology, Erasmus MC Cancer Institute, Doctor Molewaterplein 40, 3015 GD Rotterdam, The Netherlands; r.feelders@erasmusmc.nl

**Keywords:** adrenal metastasis, adrenalectomy, treatment management, patient-tailored treatment

## Abstract

**Simple Summary:**

Adrenal glands are common dissemination sites for metastases of various solid tumors. The rapid development of new treatment strategies, such as targeted therapy and immunotherapy for different cancer types, has led to increased metastatic adrenalectomies. Therefore, clear communication between oncologists and adrenal gland specialists has become increasingly important to outweigh surgical risks versus oncological advantages of adrenalectomies in these patients. This study assesses trends in diagnosis, type of surgery, and short-term and long-term surgical outcomes of patients who underwent metastatic adrenalectomy. We included a total of 95 patients with an adrenal metastasis of non-adrenal primary tumors, most often colorectal or lung cancer, who underwent (minimal invasive) adrenalectomy. 37.9% of the patients experienced one or more complications after adrenalectomy. Within our patient cohort, an increased demand for metastatic adrenalectomy was observed over the past years, which might be associated with the rise of targeted- and immunotherapy. Our data aims to assist multidisciplinary teams with weighing the pros and cons of resection of the metastasized adrenal gland of cancer patients.

**Abstract:**

The adrenal glands are common dissemination sites for metastasis of various solid tumors. Surgical treatment is often recommended because targeted therapies and immunotherapy are frequently ineffective for adrenal metastasis. We report the experience with short-term and long-term surgical outcomes of patients undergoing surgery for adrenal metastasis in two hospitals. A retrospective, multicenter study was performed to analyze patient characteristics, tumor-related data, perioperative outcomes, and oncological outcomes. Postoperative complications that occurred within 30 days were scored according to the Clavien Dindo classification. Metastatic adrenalectomy was performed in 95 patients. We observed an increase from an average of 3 metastatic adrenalectomies per year between 2001–2005 to 10 between 2015–2019. The most frequent underlying malignancies were colorectal and lung cancer. In 55.8%, minimal invasive adrenalectomy was performed, including six conversions to open surgery. A total of 37.9% of patients had postoperative complications, of which ileus or gastroparesis, wound problems, pneumonia, and heart arrhythmias were the most occurring complications. Improved cancer care has led to an increased demand for metastatic adrenalectomy over the past years. Complication rates of 37.9% are significant and cannot be neglected. Therefore, multidisciplinary teams should weigh the decision to perform metastatic adrenalectomy for each patient individually, taking into account the drawbacks of the described morbidity versus the potential benefits.

## 1. Introduction

Due to a rich blood supply [1], the adrenal glands are common dissemination sites for cancer metastases of various solid tumors of different tumor entities [2]. Large autopsy studies revealed the presence of adrenal metastases in 14% of the patients with colorectal cancer [3]. For other cancer types, the percentage of patients with adrenal metastases is even higher [4], with 42% for lung cancer and 58% for breast cancer [5]. Most of the detected adrenal metastases are one of the multiple sites of the metastasized disease. Adrenal metastases are increasingly detected due to better-quality imaging techniques, standardized routine imaging follow-up [6], and improved cancer care over the years, including better treatment strategies with prolonged overall survival.

Unfortunately, upcoming treatment strategies such as immunotherapy, targeted therapies, and chemotherapy have shown to frequently be ineffective for treating adrenal metastases [7,8,9]. Although the reason for this treatment immunity remains unknown, it highlights the importance of a safe and well weighed surgical treatment strategy. Several studies suggested a survival benefit from adrenalectomy for (oligo)metastatic disease [10,11,12,13,14,15]. Next to this, surgical techniques have improved in the last decades by introducing minimally invasive techniques such as lateral transperitoneal adrenalectomy (LTA) and retroperitoneoscopic posterior adrenalectomy (RPA). These approaches have proven feasible and safe and are associated with less morbidity than open adrenalectomy [2,15,16,17,18,19,20] while preserving equal oncological outcomes [18,20].

For this reason, current guidelines published by the American Association of Clinical Endocrinologists (AACE) and American Association of Endocrine Surgeons (AAES) recommend adrenalectomy for adrenal metastasis [21], and even minimally invasive adrenalectomy is no longer controversial [22]. Despite the guideline’s recommendation, it is often hard for disease-specific multidisciplinary tumor boards to decide which patient could withstand surgery and benefit from adrenalectomy for metastatic disease. Hence, it is of paramount importance to assess surgical complications of metastatic adrenalectomy and aid in selecting proper patients for surgery.

Therefore, our objective was to assess trends in metastatic adrenal surgery and show surgical complication rates and short-term and long-term outcomes.

## 2. Materials and Methods

### 2.1. Study Design

We retrospectively reviewed the medical records of patients undergoing metastatic adrenalectomy in two academic centers in the Netherlands (University Medical Center Groningen (UMCG) and Erasmus Medical Center Rotterdam (EMC)) between September 2001 and September 2020. The Institutional Review Boards of both participating centers have approved this study.

### 2.2. Inclusion and Exclusion Criteria

We included patients after adrenalectomy for histologically proven adrenal metastasis, regardless of synchronous or metachronous presentation. Patients with extra-adrenal metastatic disease concurrent with the adrenal metastasis were only included if treatment consisted of a step-up strategy with a curative intention for all metastatic sites. Patients were excluded if (1) no adrenalectomy was performed, (2) definitive pathological diagnosis did not confirm metastasis within the adrenal gland.

### 2.3. Data Collection

Data were obtained by reviewing the medical records of the included patients in the Electronic Patient File system. Data was collected from each hospital by a member of the local research team (M.J.H.M., C.L.V. and A.J.S.). Data were checked for accuracy by an independent researcher. We collected demographic variables (patient age, gender, ASA score), tumor-related data, and patient outcomes. Tumor-related data consisted of primary tumor type, tumor size, and laterality. Tumor size was extracted from the preoperative imaging, on which the maximum tumor diameter per adrenal metastasis was measured. In addition, tumor size and surgical margins were extracted from pathology reports. A radical surgical margin was defined as no residual tumor (R0 resection) and a non-radical resection as residual tumor (R1 or R2 resection) described by a dedicated pathologist. Adrenal metastasis was considered synchronous if the adrenal metastasis was detected within six months after diagnosing the primary malignancy. If the adrenal metastasis was detected after six months, the adrenal metastasis was considered metachronous. Perioperative variables consisted of date and type of surgery, open or laparoscopic surgery, conversion, surgical approach (LTA or RPA), surgical resection margins, duration of hospital stay, and complications. Complications within 30 days postoperatively were scored using the Clavien Dindo classification [16]. Patients were followed up until death or the date of the last follow-up. In case a patient had died, the cause of death was registered. In case patients were lost to follow-up, the reason for this was noted. Our primary outcome was the occurrence of complications after adrenalectomy. Secondary outcomes included patient time to death and completeness of metastasis resection.

### 2.4. Analysis

Categorical data are presented as frequency or percentage. Continuous data are presented as median with interquartile range (IQR) and are evaluated using the Mann-Whitney *U* test. Statistical analyses are done using GraphPad Prism (version 9.0, GraphPad Software Inc., San Diego, CA, USA).

## 3. Results

### 3.1. Patient Characteristics

**Figure 1:**
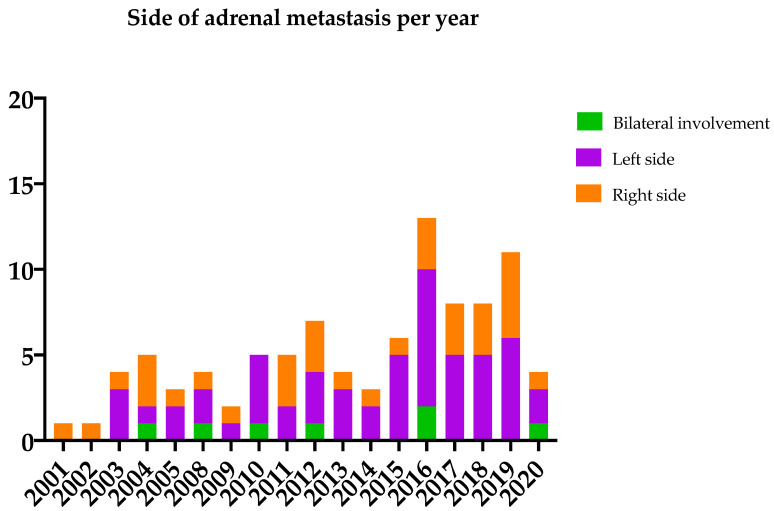
Location of adrenal metastasis.

During the study period, we included a total of 95 patients who underwent metastatic adrenalectomy, of which 20 patients with synchronous metastasis and 75 with metachronous metastasis. The most frequent underlying malignancies were colorectal cancer (27%) and lung cancer (27%), followed by melanoma (17%), renal cell cancer (7%), and breast cancer (4%) (Table 1). The cohort was predominantly male (64%) and the median age at surgery was 62 years (IQR 13 years). The left side was the most common side for unilateral adrenal involvement (*n* = 55 patients) compared to the right side (*n* = 33). Bilateral metastases were found in 7 patients (Figure 1). Data regarding tumor location, e.g., right arm or left lung, was available in 45 patients with different tumor types, except colorectal cancer. We found no association between the side of the primary tumor and the side of the adrenal metastasis. In 11 patients with different tumor types, the side of adrenal metastases was similar to the side of the primary tumor. Concomitant metastases in other organs were present in 15 patients, of which 8 patients were primarily diagnosed with the synchronous metastasized disease. The most common other locations of metastasized disease included liver and lung, for which surgery was performed. The median diameter of the adrenal metastases was 33 mm on preoperative imaging and 35 mm on pathological reports. Metastasis size did not differ across the different tumor types (Table 2). We refer to Table S1 for additional patient characteristics and Figure S1 for the tumor type per year distribution.

Ten patients underwent a more extensive resection/surgery besides an adrenalectomy alone. These procedures varied from excision of cutaneous melanoma to hepatic and colonic resections. In two patients, extended surgery was performed due to tumor invasion in surrounding organs. The patient characteristics are more extensively described in Table S2.

### 3.2. Surgical Procedures

**Figure 2:**
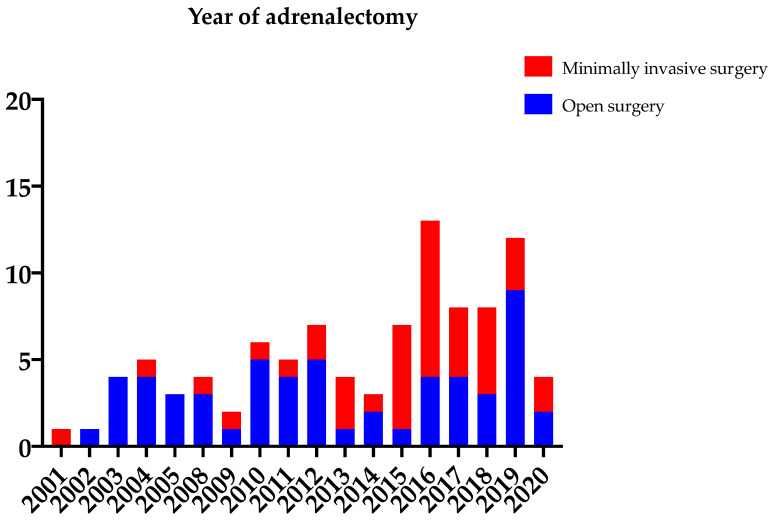
The number of adrenalectomies for adrenal metastasis performed per year per type of surgery.

We observed an increase from an average of 3 metastatic adrenalectomies per year between 2001–2005 to 10 between 2015–2019, as is presented in Figure 2. The use of minimally invasive surgical techniques predominantly increased over the last years. In total, minimal invasive adrenalectomy was performed in 55.8% of the patients (39 lateral transperitoneal adrenalectomies and 14 retroperitoneoscopic posterior adrenalectomies), including six conversions to open surgery (Table 1). One reason for conversion was tumor size, respectively 70 mm on preoperative imaging. The other patients undergoing minimally invasive surgery had a maximum tumor size of 54 mm. The median size of adrenal metastasis (28 mm, IQR 20) removed by minimally invasive surgical techniques was significantly (*p* = 0.010) smaller compared to median tumor size in patients undergoing open adrenalectomy (40 mm, IQR 73).

In all 95 patients, the adrenal specimen was subjected to pathological examination. The resection margin status, radical versus non-radical resection margin, was available in 72 patients (75.8%). Radical resection was performed in 70.8% of the patients, as stated in Table 1. The percentage of radical resections was slightly higher for open adrenalectomy but did not differ significantly across the different surgical techniques (78% for open adrenalectomy, 65% for minimally invasive surgery). Radical resection was achieved in 100% of the patients with metastasis from breast cancer or renal cancer. However, radical metastasectomy was less likely to be achieved in colorectal metastasis (63.2%) and lung metastasis (65.2%).

### 3.3. Short-Term and Long-Term Outcomes

The median hospital stay was 6.8 days. There were no intraoperative deaths, and two patients died within 30-days postoperatively. One patient with metastasized colorectal cancer underwent a combined adrenalectomy with liver segmental resection because of liver metastasis. Postoperatively, this patient developed biliary peritonitis with refractory septic shock, due to which the patient died 5 days postoperatively (Table S2). The other patient suffered from concomitant lung and liver metastasized gastric cancer. Preoperatively, the patient was diagnosed with pulmonary aspergillus, which caused respiratory insufficiency postoperatively, with limited treatment options due to liver failure secondary to liver metastases. Out of the 95 patients, 36 patients (37.9%) experienced one or more complications (Table 3). Two or more complications were observed in 9 patients (9.5%) of which two patients underwent adrenalectomy in combination with another surgery. A total of 54 postoperative complications occurred, of which the majority consisted of Clavien Dindo grade 2 complications (*n* = 32). The most frequent complications were either ileus or gastroparesis (*n* = 7), wound problems including infection or hematoma (*n* = 6), pneumonia (*n* = 5), and heart arrhythmias (*n* = 5). An overview of all documented complications in our patient series is shown in Table 4. Most complications were observed in patients undergoing adrenalectomy for metastasis of colorectal cancer (Table 3). There was a difference in complication rate between open adrenalectomy (47.6%) and minimal invasive adrenalectomy (30.2%). We did not find a difference between the number of complications in patients with a radical resection (*n* = 15) and patients with a non-radical resection (*n* = 12).

A total of 53 patients (55.8%) were deceased during follow-up. The majority, 67.9%, of the deceased patients underwent adrenalectomy for metachronous adrenal metastasis. In contrast, 55.5% of the patients with lung cancer underwent adrenalectomy for synchronous metastasis. The median time to death was calculated using data of 52 patients due to the absence of a deceased date of one patient. The median time to death after adrenalectomy for all deceased patients was 20.2 months. Median time to death varied widely across different primary tumor types, ranging from 29.97 months (*n* = 17) for colorectal cancer to 8.49 months and 7.96 months for lung cancer (*n* = 17) and melanoma (*n* = 3) respectively (Table 5).

## 4. Discussion

With the increased possibilities for both detection and treatment of metastatic disease, surgical treatment for (oligo)metastatic disease in the adrenal gland has become more accepted over the last years. We, therefore, evaluated clinical characteristics and surgical outcomes of patients who underwent metastatic adrenalectomy in two academic centers in the Netherlands. In these centers, we observed an increase in the frequency of adrenalectomy for the metastatic disease over the years. Metastatic adrenalectomy was primarily performed in colorectal cancer, lung cancer, and patients with melanoma. Despite the emergence of minimally invasive techniques such as lateral transperitoneal adrenalectomy and retroperitoneoscopic posterior adrenalectomy associated with less morbidity, complication rates cannot be neglected. At least one complication occurred in up to 37.9% of the patients undergoing metastatic adrenalectomy. This complication rate underlines the need to carefully outweigh the benefits against the risk of doing more damage to patients with disseminated disease. The postoperative complication rate and description of common complications can be used to inform the patient about surgical treatment.

In our study, the increase in metastatic adrenalectomy performed each year could be explained by improved cancer care [6] but also by the improved surgical techniques with advantages for patients such as shorter duration of hospital stay and lower complication rates [16,17]. Lung cancer and colorectal cancer were the most frequent underlying malignancy of adrenal metastasis within our patient cohort. Lung cancer as the origin of the metastasis seems to be concordant with the literature. However, colorectal cancer has not previously been described as one of the most common origins [23,24,25,26]. A possible reason for the found percentage of primary colorectal cancers could be explained by the fact that the Netherlands has one of the highest incidences of colorectal cancer worldwide [27]. The nationally organized screening program also has the highest participation rates leading to more patients diagnosed with colorectal cancer. However, surveillance is more extensive compared to other countries due to the higher incidence. Surveillance includes more imaging follow-ups detecting possible metastasis [28].

In our patient cohort, the majority of patients underwent surgery for metachronous adrenal metastasis. This finding contradicts the study of Lam et al., where 67% of the patients were diagnosed with adrenal metastasis synchronously with the primary tumor. That study described diagnosing metastatic adrenal disease upon histological examination, including autopsy reports and biopsies. Only 21 patients underwent adrenalectomy [29]. In another large cohort of patients undergoing adrenalectomy for metastatic disease, 63% of the patients were diagnosed with metachronous metastatic disease [26]. Studies showed conflicting data regarding the laterality of adrenal metastasis. Most studies described predominantly unilateral adrenal [13,26,30,31], although some studies reported comparable uni- and bilateral involvement [32] and few studies even reported mostly bilateral metastasis [33].

Our cohort’s complication rates after metastatic adrenalectomy are comparable to different cohorts in the literature [10,23]. Literature shows a wide range of complication rates from zero to 63% [18]. In this study, we found a higher complication rate after open adrenalectomy. A possible explanation for this might be that patients’ characteristics of the patients undergoing open adrenalectomy are less favorable, as patients might not be suitable for minimally invasive techniques due to previous surgeries, size of adrenal metastasis, or comorbidities. Furthermore, our study confirmed the safety of laparoscopic techniques for metastatic adrenalectomy and demonstrated the increased performance of minimally invasive techniques [34,35,36]. Previous research has demonstrated that minimally invasive surgery can be safely performed for lesions even beyond a diameter of 60 mm. Laparoscopy in larger tumors was, however, in these studies associated with a longer operation time and a higher risk of conversion or intra-operative complications [36]. Based on these data and our findings, we recommend (if technically possible) minimally invasive surgery for (oligo)metastasis for all metastasis <60 mm. For larger tumors, it is important to outweigh the surgical risks of an open procedure in patients with metastasized cancer with great variability in condition among patients against the intraoperative risks of laparoscopy in these patients. Consistent with the literature, this research found that patients with renal cell cancer have the longest median time to death after surgery, which was also described by Ramsingh et al. [23].

The current study has several limitations. First, this retrospective study could be subject to selection bias despite all attempts to obtain complete medical information. Due to the retrospective nature of this study, not all obtained data from the Electronic Patient File was noted in a systematic way leading to missing data and the inability to analyze all data for all patients. Second, the relatively small sample size of patients per tumor type hampers the interpretation of the data for each tumor type specifically as patient and tumor characteristics vary widely. This study included all tumor types, therefore the separate description of tumor characteristics and outcomes per tumor type should be interpreted with caution, especially for those with a lower incidence.

Furthermore, as all patients in our cohort underwent metastatic adrenalectomy, we cannot compare their outcomes to patients with adrenal metastases who did not have surgery. However, it was not the aim of the study to review the clinical and tumor characteristics of all patients with adrenal metastasis and draw a clear conclusion regarding the oncological outcome. This study’s results mainly describe the current situation regarding metastatic adrenalectomy and offer a perspective on the current clinical practice, rather than providing guidelines for the surgical management of adrenal metastasis and advising for or against surgical treatment. With our results, the multidisciplinary tumor boards have a better perspective of short-term and long-term outcomes.

Additional research is necessary to provide a clear conclusion on which patients benefit from surgery. This research should include all patients, resected or not, with adrenal metastasis. To analyze for which tumor type metastatic adrenalectomy is beneficial, a prospective multicenter study should ideally include the required number of patients with adrenal metastasis originating from the different tumor types. However, due to the low incidence of (oligo)metastatic disease in the adrenal gland, the required inclusion period for such a study is expected to be quite long. Next to this, the study will be subject to a risk of selection bias as the study will be associated with variability in the behavior of the primary tumor. This overview of patient characteristics and outcomes of patients undergoing metastatic adrenalectomy offers an insight into the current daily practice in two academic hospitals in the Netherlands. It may aid multidisciplinary tumor boards in the discussion for an individually tailored treatment strategy. This study shows the importance of an individual approach for all patients with adrenal metastasis.

We reported the experience of two academic centers performing adrenalectomy for adrenal metastases. The number of metastatic adrenalectomies increased over time and metastatic adrenalectomy was mainly performed in patients with a primary colorectal or lung cancer and melanoma. A significant complication rate should be considered when deciding whether or not to perform surgery in patients with disseminated disease, often during palliative care. A multidisciplinary tumor board should discuss an individually tailored treatment strategy for each patient.

## 5. Conclusions

Improved cancer care has led to an increased demand for metastatic adrenalectomy over the past years. Complication rates of 37.9% are significant and cannot be neglected.

Therefore, multidisciplinary teams should weigh the decision to perform metastatic adrenalectomy for each patient individually, taking into account the drawbacks of the described morbidity versus the potential benefits.

## Figures and Tables

**Table 1:** Tumor type distribution. Type of surgery, the onset of adrenal metastasis, and resection margin per tumor type (number of patients and percentages).

Primary Tumor	Patients *n* (%)	Type of Surgery Open versus Minimally Invasive *n* (%) versus *n* (%)	Onset of Adrenal Metastasis Synchronous versus Metachronous *n* (%) versus *n* (%)	Concomitant Metastases *n* (*n* Synchronous versus *n* Metachronous Disease)	Resection Margin Radical versus Non-Radical *n* (%) versus *n* (%)
All adrenal metastasis	95 (100)	42 (44.2) versus 53 (55.8)	20 (21.1) versus 75 (78.9)	15 (8 versus 7)	51 (70.8) versus 21 (29.2) ^1^
Colorectal cancer	25 (27)	14 (56.0) versus 11 (44.0)	2 (8.0) versus 23 (92)	3 (0 versus 3)	12 (63.2) versus 7 (36.8)
Lung cancer	25 (27)	6 (24.0) versus 19 (76.0)	11 (44.0) versus 14 (56.0)	4 (4 versus 0)	15 (65.2) versus 8 (34.8)
Melanoma	16 (17)	8 (50.0) versus 8 (50.0)	2 (12.5) versus 14 (87.5)	4 (1 versus 3)	9 (75.0) versus 3 (25.0)
Renal cell cancer	7 (7)	4 (57.1) versus 3 (42.9)	0 (0) versus 8 (100)	N/A	6 (100.0) versus 0 (0.0)
Breast cancer	4 (4)	0 (0.0) versus 4 (100.0)	1 (25.0) versus 3 (75.0)	1 (1 versus 0)	2 (100.0) versus 0 (0.0)
Other cancer types	18 (19)	10 (55.5) versus 8 (44.5)	5 (27.8) versus 13 (72.2)	3 (2 versus 0)	6 (75.0) versus 2 (25.0)

Abbreviations: N/A = not applicable; ^1^ Information about resection margin available in 72 patients.

**Table 2:** Tumor size per tumor type.

Radiological			Histological	
Primary Tumor	Adrenal Metastasis (*n*)	Maximum Tumor Diameter (mm) Median (IQR)	Adrenal Metastasis (*n*)	Maximum Histological Tumor Diameter (mm) Median (IQR)
All adrenal metastasis	77	33 (21)	83	35 (36)
Colorectal cancer	21	32 (17)	25	39 (38)
Lung cancer	18	40 (18.25)	19	40 (37)
Melanoma	13	36 (27)	12	38.5 (41)
Renal cell cancer	8	34 (14.75)	7	28 (15)
Breast cancer	3	28 (34)	3	28 (28)
Other cancer types	14	22 (8.25)	17	35 (36)

Abbreviations: IQR = Interquartile range.

**Table 3:** The complication rate after metastatic adrenalectomy per tumor type.

Primary Tumor	Patients *n* (%)	Complications *n* (% per Tumor Type)
All adrenal metastasis	95 (100)	36 (100)
Colorectal cancer	25 (27)	10 (40)
Lung cancer	25 (27)	8 (32)
Melanoma	16 (17)	5 (31.25)
Renal cell cancer	7 (7)	4 (57.14)
Breast cancer	4 (4)	2 (50)
Other cancer types	18 (19)	7 (38.89)

**Table 4:** Description of complications observed in patients after metastatic adrenalectomy.

Complication	Patients *n (%)*
All complications	54 (100)
Ileus/gastroparesis	7 (13.0)
Wound problems	6 (11.1)
Pneumonia	5 (9.3)
Heart arrhythmias	5 (9.3)
Delirium	4 (7.4)
Fluid overload/edema	3 (5.6)
Electrolyte imbalance	3 (5.6)
Urinary tract infection	2 (3.7)
Bladder retention	2 (3.7)
Anemia	2 (3.7)
Bleeding	2 (3.7)
Postoperative pain	2 (3.7)
Septic shock	1 (1.8)
Bowel perforation	1 (1.8)
Bile leakage	1 (1.8)
Pneumatic embolism	1 (1.8)
Pneumothorax	1 (1.8)
Decubitus	1 (1.8)
Abscess	1 (1.8)
Hypertension	1 (1.8)
Diabetes de novo	1 (1.8)
Constipation	1 (1.8)
Fever	1 (1.8)

**Table 5:** Mortality rate and median time to death after metastatic adrenalectomy per tumor type.

Primary Tumor	Patients *n* (%)	Deceased Patients *n* (%)	Onset of Adrenal Metastasis Synchronous vs. Metachronous *n* versus *n*	Median Time to Death after MA Months (IQR)
All adrenal metastasis	95 (100)	53 (100)	16 versus 37	20.2 (24.9) ^1^
Colorectal cancer	25 (27)	17 (68)	2 versus 15	29.97 (25.17)
Lung cancer	25 (27)	18 (72)	10 versus 8	8.49 (15.88) ^2^
Melanoma	16 (17)	3 (18.75)	1 versus 2	7.96 (10.06)
Renal cell cancer	7 (7)	3 (42.86)	0 versus 3	40.37 (71.29)
Breast cancer	4 (4)	1 (25)	0 versus 1	46.82 (N/A)
Other cancer types	18 (19)	11 (57.89)	3 versus 8	22.08 (24.57)

Abbreviations: IQR = Interquartile range, MA = metastatic adrenalectomy; ^1^ Calculations based on 52 patients; ^2^ calculations based on 17 patients.

## Data Availability

The data presented in this study are available on request from the corresponding author.

## Supplementary Materials

The following are available online at https://www.mdpi.com/article/10.3390/cancers14010156/s1, Figure S1: Distribution of tumor types per year, Table S1: Additional patient characteristics per tumor type (number of patients and percentages), Table S2: Patient characteristics of patient undergoing adrenalectomy combined with another procedure.
