# Supplementary material for: Outcomes after Surgical Treatment of Metastatic Disease in the Adrenal Gland; Valuable for the Patient?
Source: Cancers (Basel). 2021 Dec 29;14(1):156. doi: 10.3390/cancers14010156 (PMC8750225; doi:10.3390/cancers14010156)
Supplement: Supplementary file 1 [file cancers-14-00156-s001.zip › cancers-1502380-supplementary.pdf]

*Supplementary Materials*

# Outcomes after Surgical Treatment of Metastatic Disease in the Adrenal Gland; Valuable for the Patient?

**Madelon J.H. Metman, Charlotte L. Viëtor, Auke J. Seinen, Annika M.A. Berends, Patrick H.J. Hemmer, Michiel N. Kerstens, Richard A. Feelders, Gaston J.H. Franssen, Tessa M. van Ginhoven and Schelto Kruijff**

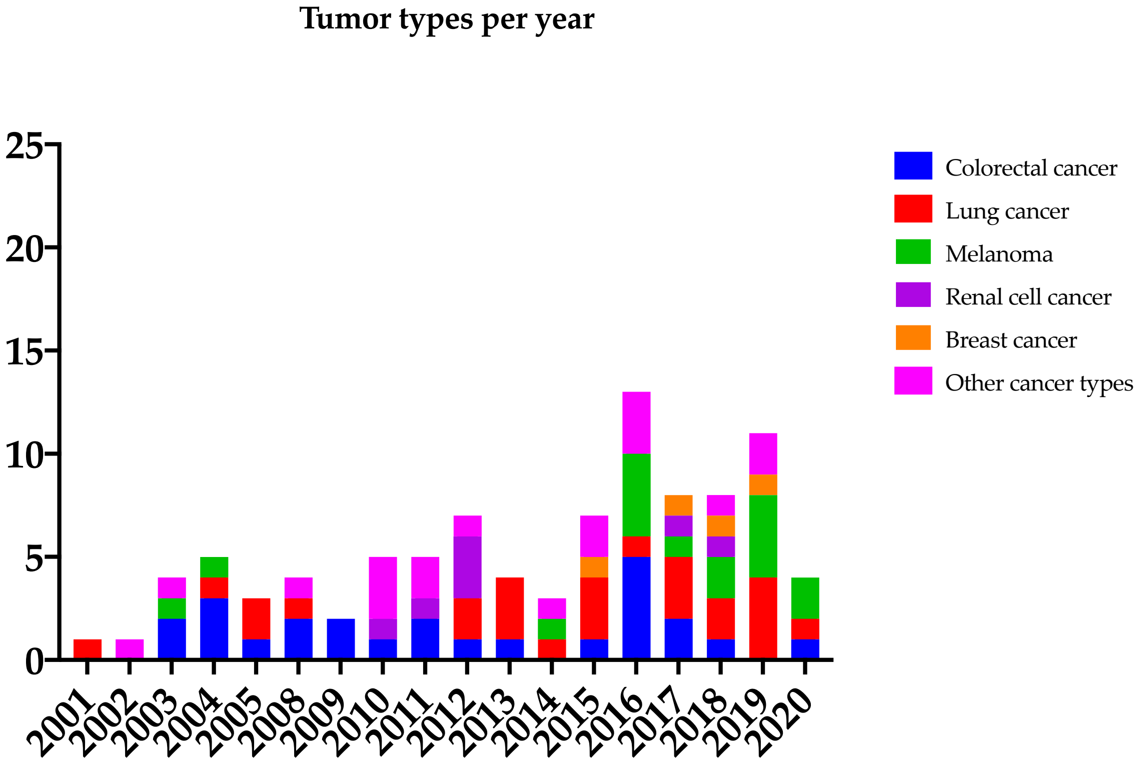


**Figure S1.** Distribution of tumor types per year.

**Table S1.** Additional patient characteristics per tumor type (number of patients and percentages).

| Cancer Type | Patients *n* (%) | Sex Male versus female *n* (%) versus *n* (%) | Age Years (IQR years) | ASA classification Classification *n* (%) |
|---|---|---|---|---|
| Total | 95 (100) | 61 (64.21) versus 34 (35.79) | 63 (12.5) | 1—1 (1.05)<br>2—62 (65.25)<br>3—24 (25.26)<br>Unknown—8 (8.42) |
| Colorectal cancer | 25 (27) | 21 (84) versus 4 (16) | 62 (12.5) | 1—N/A<br>2—19 (76)<br>3—2 (12)<br>Unknown—4 (16) |
| Lung cancer | 25 (27) | 10 (40) versus 15 (60) | 61 (12) | 1—N/A<br>2—13 (52)<br>3—10 (40)<br>Unknown—2 (8) |

| | | | | |
|---|---|---|---|---|
| Melanoma | 16 (17) | 13 (81.25) versus 3 (18.75) | 61.5 (20.75) | 1—1 (6.25)<br>2—12 (75)<br>3—2 (12.5)<br>Unknown—1 (6.25) |
| Renal cell cancer | 7 (7) | 3 (42.56) versus 4 (57.14) | 70.0 (13.0) | 1—N/A<br>2—5 (71,73)<br>3—2 (28.57)<br>Unknown—N/A |
| Breast cancer | 4 (4) | 0 (0) versus 4 (100) | 68.5 (23.25) | 1—N/A<br>2—3 (75)<br>3—1 (25)<br>Unknown—N/A |
| Other cancer types | 18 (19) | 4 (22.22) versus 14 (77.78) | 65 (12.5) | 1—N/A<br>2—10 (55.55)<br>3—7 (38.89)<br>Unknown—1 (5.56) |

Abbreviations: IQR = Interquartile range, N/A= not applicable.

**Table S2.** Patient characteristics of patient undergoing adrenalectomy combined with another procedure.

| No. | Primary Tumor | Procedure in Conjunction with Adrenalectomy | Type of Surgery | Onset of Adrenal Metastasis | Complication(s) |
|---|---|---|---|---|---|
| Patient 1 | Colorectal cancer | Liver segment resection | Laparotomy | Metachronous | Death, septic shock, delirium, heart arrhythmias |
| Patient 2 | Colorectal cancer | Left hemicolectomy | Laparotomy | Synchronous | Diabetes de novo |
| Patient 3 | Lung cancer | Spleen and kidney resection due to tumor invasion | Laparotomy | Metachronous | Pneumonia |
| Patient 4 | Lung cancer | Lobectomy | Laparotomy | Synchronous | N/A |
| Patient 5 | Melanoma | Left hemicolectomy and small bowel resection | Laparotomy | Synchronous | N/A |
| Patient 6 | Melanoma | Wedge resection of stomach | Laparotomy | Metachronous | Gastroparesis |
| Patient 7 | Melanoma | Liver segment resection due to tumor invasion | Laparotomy | Metachronous | N/A |
| Patient 8 | Melanoma | Resection primary tumor right leg | Laparoscopy | Synchronous | N/A |
| Patient 9 | Renal cell carcinoma | Right hemihepatectomy | Laparotomy | Metachronous | Bile leakage, delirium, hypernatremia, hypokalemia |
| Patient 10 | Breast cancer | Modified radical mastectomy | Laparoscopy | Synchronous | Anemia |
| Patient 11 | Liver cancer | Distal pancreatectomy and splenectomy due to tumor invasion | Laparotomy | Metachronous | N/A |
| Patient 12 | Nonseminomatous germ cell tumor | Orchidectomy and lymph node dissection | Laparotomy | Synchronous | N/A |

Abbreviations: N/A= not applicable.

© 2021 by the authors. Licensee MDPI, Basel, Switzerland. This article is an open access article distributed under the terms and conditions of the Creative Commons Attribution (CC BY) license (http://creativecommons.org/licenses/by/4.0/).
